# Exploring the Antiviral Potential of Esters of Cinnamic Acids with Quercetin

**DOI:** 10.3390/v16050665

**Published:** 2024-04-24

**Authors:** Valeria Manca, Annalisa Chianese, Vanessa Palmas, Federica Etzi, Carla Zannella, Davide Moi, Francesco Secci, Gabriele Serreli, Giorgia Sarais, Maria Vittoria Morone, Massimiliano Galdiero, Valentina Onnis, Aldo Manzin, Giuseppina Sanna

**Affiliations:** 1 Microbiology and Virology Unit, Department of Biomedical Sciences, University of Cagliari, Cittadella Universitaria, 09042 Monserrato, Italy; vanessa.palmas@unica.it (V.P.); aldomanzin@unica.it (A.M.); 2 Department of Experimental Medicine, University of Study of Campania “Luigi Vanvitelli”, Via Costantinopoli 16, 80138 Napoli, Italy; annalisa.chianese@unicampania.it (A.C.); carla.zannella@unicampania.it (C.Z.); mariavittoriamorone@gmail.com (M.V.M.); massimiliano.galdiero@unicampania.it (M.G.); 3 Biology and Genetic Unit, Department of Biomedical Sciences, University of Cagliari, Cittadella Universitaria, 09042 Monserrato, Italy; federicaetzi@gmail.com; 4 Department of Life and Environmental Sciences, University of Cagliari, Cittadella Universitaria, 09042 Monserrato, Italy; davide.moi2@gmail.com (D.M.); gsarais@unica.it (G.S.); vonnis@unica.it (V.O.); 5 Department of Chemical and Geological Sciences, University of Cagliari, Cittadella Universitaria, 09042 Monserrato, Italy; fsecci@unica.it; 6 Pathology Unit, Department of Biomedical Sciences, University of Cagliari, Cittadella Universitaria, 09042 Monserrato, Italy; gabriele.serreli@unica.it

**Keywords:** quercetin, cinnamic acid esters, flavonoids, Coronaviruses, antivirals

## Abstract

Severe acute respiratory syndrome-related Coronavirus 2 (SARS-CoV-2) has infected more than 762 million people to date and has caused approximately 7 million deaths all around the world, involving more than 187 countries. Although currently available vaccines show high efficacy in preventing severe respiratory complications in infected patients, the high number of mutations in the S proteins of the current variants is responsible for the high level of immune evasion and transmissibility of the virus and the reduced effectiveness of acquired immunity. In this scenario, the development of safe and effective drugs of synthetic or natural origin to suppress viral replication and treat acute forms of COVID-19 remains a valid therapeutic challenge. Given the successful history of flavonoids-based drug discovery, we developed esters of substituted cinnamic acids with quercetin to evaluate their in vitro activity against a broad spectrum of Coronaviruses. Interestingly, two derivatives, the 3,4-methylenedioxy 6 and the ester of acid 7, have proved to be effective in reducing OC43-induced cytopathogenicity, showing interesting EC50s profiles. The ester of synaptic acid 7 in particular, which is not endowed with relevant cytotoxicity under any of the tested conditions, turned out to be active against OC43 and SARS-CoV-2, showing a promising EC_50_. Therefore, said compound was selected as the lead object of further analysis. When tested in a yield reduction, assay 7 produced a significant dose-dependent reduction in viral titer. However, the compound was not virucidal, as exposure to high concentrations of it did not affect viral infectivity, nor did it affect hCoV-OC43 penetration into pre-treated host cells. Additional studies on the action mechanism have suggested that our derivative may inhibit viral endocytosis by reducing viral attachment to host cells.

## 1. Introduction

Coronaviruses (CoVs) are a family of enveloped, positive-sense single-stranded RNA viruses. The family Coronaviridae is found within the order Nidovirales, suborder Coronavirineae, and is distinguished in the subfamily Orthocoronavirinae, which includes four genera: alphacoronavirus, betacoronavirus, gammacoronavirus and deltacoronavirus. The first two genera infect only mammalian species, while the others have a wider host range which also includes avian species [1].

The human coronaviruses HCoV-229E and HCoV-OC43, together with the more recently discovered HCoV-NL63 and HCoV-HKU1, cause mild respiratory infections associated with “common cold” symptoms, while the severe acute respiratory syndrome coronaviruses (SARS-CoV and SARS-CoV-2) and Middle East respiratory syndrome (MERS-CoV) are highly pathogenic in humans, infecting both bronchial epithelial cells and upper airway cells and [1] pneumocytes.

SARS-CoV-2 belongs to the Severe Acute Respiratory Syndrome-related coronavirus species in the subgenus Sarbecovirus. Since 2019 it has spread rapidly in the human population after likely spillover from bats and intermediate hosts, causing the COVID-19 pandemic known as the “coronavirus disease 2019”. Since the first case occurred on 17 November 2019, in Wuhan, the World Health Organization has confirmed more than 700 million cases among more than 200 countries, and more than 7 million deaths [2].

This pathogen affects the upper and lower respiratory tracts, has an efficient human-to-human transmission and causes typical clinical manifestations such as fever, dry cough, fatigue and in some cases even serious problems such as respiratory failure and death, as well as systemic involvement due to the massive inflammatory syndrome caused by the virus, especially in the elderly and in patients with comorbidities [3].

**Figure 1:**
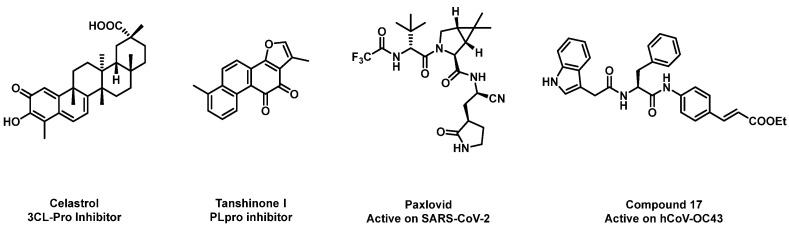
Natural and synthetic compounds active on SARS-CoV-2 and hCoV-OC43 [5,6,7,8].

To date, several compounds have been shown to be active at the different stages of the viral cycle (Figure 1) such as entry and replication, and monoclonal antibody therapy may also be used. Nevertheless, the most effective method for a long-term prevention and control strategy is vaccination: for this reason, especially in recent years, different types of vaccines have been developed, through the employment of various platforms including recombinant vectors, DNA, mRNA in lipid nanoparticles, inactivated or live attenuated [4] viruses and protein subunits.

Since the beginning of the COVID-19 pandemic, a considerable part of pharmaceutical research has been directed towards the discovery of new and effective antiviral drugs useful to treat and/or prevent the disease and to be available in case of future health emergencies. The search for potential antiviral agents has also led to the exploration of natural substances [9] and synthetic compounds that showcase great potential, biocompatibility and safety and represent, together with the re-proposal of proven antiviral molecules, a better and more practical approach to defeat the ongoing pandemic. With this purpose, several synthetic small molecules have been studied and were added to the first oral antiviral drug Paxlovid. 

Quercetin, a flavonol abundant in fruit and vegetables and widely used as a food supplement to strengthen the immune system, is also studied for its biological properties, such as antioxidant, anti-inflammatory and immunomodulatory activities. The combination of said actions make it one of the most clinically studied supplements, although most studies in the literature report controversial results on its therapeutic role regarding COVID-19. 

Several studies have highlighted the potential use of these flavonoids as antivirals [10,11,12,13], thanks to their ability to inhibit the initial stages of virus infection, interact with viral proteases and reduce inflammation caused by infection. Cinnamic acid and its derivatives have been reported for their biological activity as anti-inflammatory and anti-proliferative compounds thanks to their cellular protection properties and reduction of factors that trigger metabolic syndrome [14,15,16]. Ferulic acid possesses a variety of biological properties, such as antioxidant [17,18], anti-inflammatory [19,20], antibacterial [21], anticancer [22] and antiviral [23,24,25,26] activities and cardiovascular protective effects [27].

Sinapic acid, another cinnamic family derivative, also possesses various disease-modifying properties implicated with oxidative stress [28,29].

3,4-Dihydroxycinnamic acid, also known as caffeic acid, can affect cancer, diabetes, atherosclerosis and Alzheimer’s disease, as well as infections [30]. Moreover, substituted cinnamic acids are also studied for their antiviral activity, especially on the Zika virus and Hepatitis C virus [31,32].

In this study, we have described the first esters of cinnamic acids with quercetin, reported their activity on SARS-CoV-2 and hCoV-OC43 and shown their potential as adjuvants of approved antiviral agents.

## 2. Materials and Methods

### 2.1. Cells and Viruses

Cell lines were purchased from the American Type Culture Collection (ATCC). The absence of mycoplasma contamination was checked periodically by the Hoechst staining method. Cell lines supporting the multiplication of Coronaviruses were the following: Monkey kidney (Vero-76) [ATCC CRL 1587 Cercopithecus Aethiops], Monkey kidney (Vero C1008, clone E6) [ATCC CRL 1586 Cercopithecus Aethiops].

Human coronaviruses were: (i) Coronaviridae: Betacoronavirus strain OC43 (ATCC VR-1558), Alphacoronavirus strain 229E (ATCC VR-740), Betacoronavirus SARS-CoV-2 (strain VR PV10734) clinical isolate, kindly provided by Lazzaro Spallanzani Hospital, Rome, Italy. All experimental steps involving the SARS-CoV-2 virus were performed in a biosafety level 3 (BSL3) containment laboratory.

### 2.2. Cytotoxicity Assays

Vero-76 cells or Caco-2 (as reported in Supplementary Materials [33]) were seeded in 96-well plates at an initial density of 3 × 10^5^/mL, in Minimum Essential Medium with Earle’s salts (MEM-E), L-glutamine, 1 mM sodium pyruvate and 25 mg/L kanamycin, supplemented with 10% fetal bovine serum (FBS). Cell cultures were then incubated at 37 °C in a humidified, 5% CO2-enriched atmosphere, in the absence or presence of serial dilutions of test compounds. The test medium used for the cytotoxic assay as well as for the antiviral assay contained 1% of the appropriate serum. Cell viability was determined after 24, 72 (Quercetin and **7**) or 120 h at 37 °C by the MTT method [34].

### 2.3. Antiviral Assays

The compound’s activity against 229E, OC43 and EVA71 was based on the inhibition of virus-induced cytopathogenicity in Vero-76 cells acutely infected with a m.o.i. of 0.01. After 3 (229E), 5 (EVA71) or 5/6 (OC43) days of incubation at 35 °C or 37 °C (EVA71), cell viability was determined by the MTT method, as described previously [35]. The compound’s activity against SARS-CoV-2 was determined by plaque reduction assays in infected cell monolayers, as described previously [36].

### 2.4. Yield Reduction Assay

Vero-76 cells were inoculated with OC43 at a m.o.i. of 0.1 in a maintenance medium and tested compounds (**7**) at non-cytotoxic concentrations. Following a 2 h adsorption period at 35 °C and 5% CO_2_, the inoculum was removed and replaced with fresh medium containing the same concentration of tested compounds. After 120 h at 35 °C and 5% CO_2,_ each sample was harvested and diluted with serial passages, starting from 10^−1^ up to 10^−8^. The titer of the serial dilutions of the virus-containing supernatant was determined by the Reed and Muench method. Remdesivir and Hydroxychloroquine were used as reference compounds.

### 2.5. OC43 Virucidal Activity Assay

The title compound (20 µM) was incubated with 1 × 10^5^ TCID_50_/mL of OC43 at either 4 or 37 °C for 1 h. The mixture without a test sample was used as control. At the end of the incubation period, samples were serially diluted in media, and titers were determined on Vero-76 and Vero E6 cells at high dilutions, at which the compound was not active. Virus titers were determined by the Reed and Muench method in Vero-76 cells.

### 2.6. Cell Pretreatment Assay

Vero-76 and Vero E6 cell monolayers in 24-well plates were incubated with 20 µM concentration of compound **7** or references (100, 20, 4, 0.8 µM) for 2 h at 4 °C. After the removal of the compounds and two gentle washes, cells were infected with OC43 and SARS-CoV-2. After virus adsorption to cells, the inoculum was removed, and the cells were overlaid with medium and incubated for 4 days at 37 °C; then, finally, virus titers were determined by the Reed and Muench method and plaque assay, respectively.

### 2.7. Adsorption Assays

Vero-76 cells and E6 grown in 96- and 24-well plates were infected with OC43 and SARS-CoV-2, respectively, with an m.o.i. of 0.1, in the presence or absence of compound **7**. Multiwells were incubated for 60 min at 4 °C. Medium containing unabsorbed virus was then removed, and cells were washed twice with PBS and overlayed with the medium. Plaques were counted after 72 h of incubation at 37 °C for SARS-CoV-2, while titer reduction was determined after 144 h of incubation at 35 °C by the Reed and Muench method for OC43.

## 3. Results and Discussion

**Figure 2:**
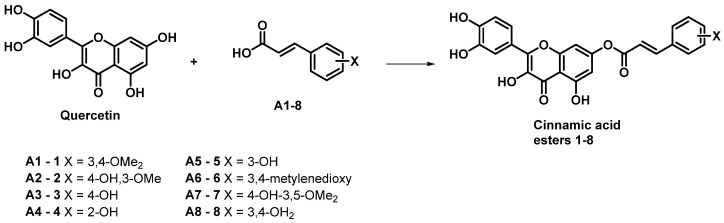
Synthesis of cinnamic acid esters.

This study explored the antiviral properties of esters of hydroxycinnamic acids with quercetin in order to investigate their potential antiviral activity on a broad spectrum of coronaviruses. The esters were prepared through a reaction between substituted cinnamic acid and quercetin, using EDCI as coupling agents in the presence of HOBt in dry acetonitrile (Figure 2) solution as described in Supplementary Materials.

Quercetin, esters, and the parent cinnamic acids were tested in cell-based assays for cytotoxicity (CC_50_), potency (EC_50_), and broad-spectrum antiviral activity against a panel of alfa and beta coronaviruses. To ascertain their selectivity against coronaviruses, the derivatives were also tested against enterovirus EVA71, another representative positive-single-stranded RNA virus (Table S2). As reported in Table 1, the esters showed an assorted profile of cytotoxicity.

Cinnamic acids showed no cytotoxicity (Table S1), except for sinapic acid (CC_50_ 44 μM. In general, the esters showed a lower level of cytotoxicity against the Vero-76 cell line than quercetin, with the sole exception of compound **2**. Compound **1** showed CC_50_ = 54.7 μM against Vero-76 and no activity on SARS-CoV-2 and OC43, while mono-substituted compound **3** showed a comparable cytotoxicity profile (CC_50_ = 60 μM) and an EC_50_ = 47.7 ± 0.32 μM aginst SARS-CoV-2. There was no evidence of activity against the beta coronavirus OC43. Moving the -OH group from 4- to 2-position (compound **4**) slightly reduced both cytotoxicity and antiviral activity, while analog ester **5** bearing the -OH in 3 position showed CC_50_ = 75 μM and an EC_50_ = 37 μM against SARS-CoV-2. 

The 3,4-methylenedioxy derivative (compound **6**) showed weak cytotoxicity (CC_50_ = 30 μM) but effectively reduced the OC43-induced cytopathogenicity, showing an EC_50_ = 2.4 ± 0.56 μM.

**Figure 3:**
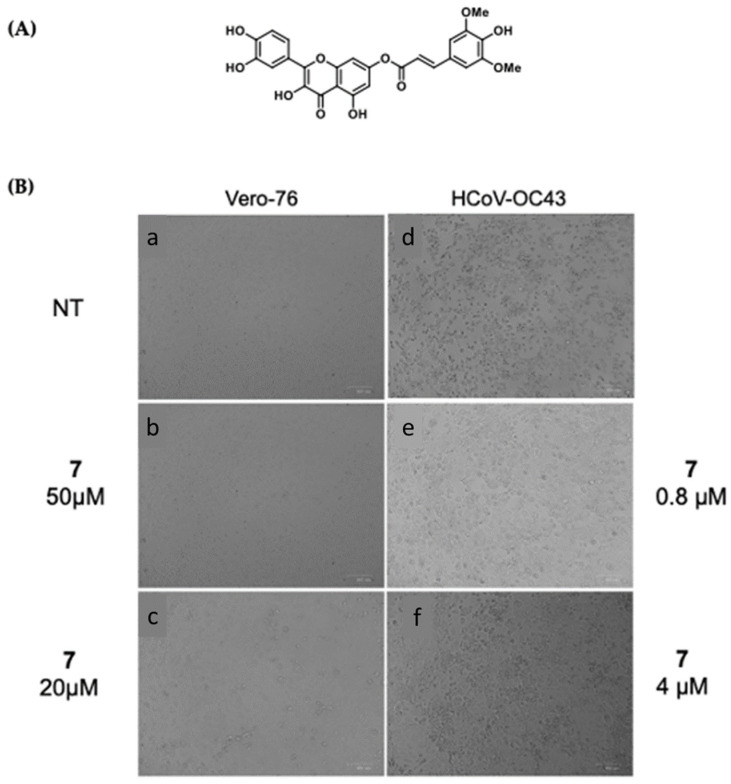
(**A**) The chemical structure of ester **7** and (**B**) validation of the effect of 7 (50, 20, 4, and 0.8 µM) on the Vero-76 HCoV-OC43-infected monolayers. Untreated control cells (**a**), treated and infected cells with 50 µM (**b**), treated and infected cells with 20 µM (**c**), treated and infected cells with 4 µM (**f**), treated and infected cells with 0.8 µM (**e**), infected cells (**d**). Pictures of cell morphology were taken at 120 h post-infection using a ZOE Fluorescent cell imager (Bio-Rad, Hercules, CA, USA) (bar size = 100 μm, magnification, 20×).

Interestingly, the ester of sinapic acid 7, which is not endowed with relevant cytotoxicity under any of the conditions tested (CC_50_ = 100 μM, Table 1 and Table S2), proved active against OC43 (Figure 3B) and SARS-CoV-2, showing a promising EC_50_ profile (EC_50_ = 6.5 ± 0.7 μM and EC_50_ = 46.6 μM, respectively).

**Figure 4:**
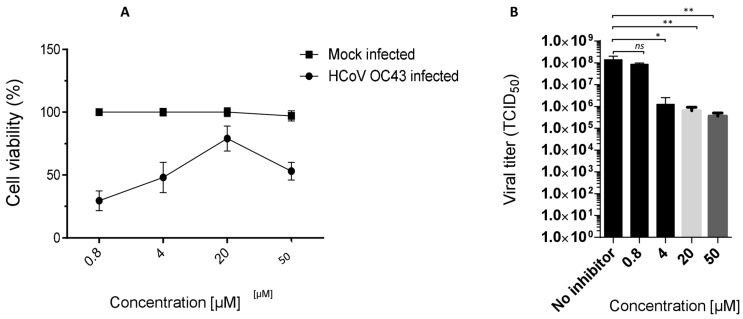
Cytotoxicity and anti OC43 activity of 7 and Yield reduction assay. (**A**) The viability of OC43-infected Vero-76 cells was estimated by MTT assay, six days after infection. The number of live cells was expressed as a percentage of mock-infected, untreated control cells. Data are expressed as means ± SD of at least three independent measurements. (**B**) The yield of infectious HCoV-OC43 viruses produced in infected Vero-76 cells treated with 7. Vero-76 cells were infected with OC43 (m.o.i. 0.1). The infected monolayers were treated with **7**, at indicated doses. Viral yields in the culture supernatant were determined by reed and Munch titration at 96 h post-infection. Statistical differences were analyzed via Student’s *t*-test, a value of *p* ≤ 0.05 was considered significant, with * = *p* ≤ 0.05, ** = *p* < 0.01, ns: not significant.

Ester **7** proved the most interesting compound of the series (Figure 4A) against the OC43 virus, with the best selectivity index (Supplementary Materials = 15).

The ester of caffeic acid (**8**) also exhibited activity against SARS-CoV-2 (EC_50_ = 26 μM) and OC43 (EC_50_ = 15 μM) followed by weak cytotoxicity on Vero-76 (CC_50_ = 70 μM). 

These results highlighted that the presence of ether groups is generally related to the best profile of antiviral activity with low toxicity. The compounds **3**–**5**, **8** bearing only hydroxy groups are endowed with low antiviral activity. However, the ether position and nature are crucial for its activity. Thus, the replacement of the 3,4-dimethoxyphenyl group of compound **1** with a 3,4-methylendioxy group led to an improvement in activity (compound **6**). The displacement of the ether groups in 3- and 5-positions and the introduction of a 4-hydroxy group afforded the best ester of series 7.

The cytotoxicity of Quercetin and **7** were evaluated, in the short (24, 72 h) and long term (144 h), also against Caco-2 cells, human colorectal adenocarcinoma cells commonly employed as a model of the intestinal epithelial barrier. No cytotoxicity was detected for Quercetin against Vero-76 cells after 24 h; however, its toxicity increased with time (after 144 h CC_50_ = 20 μM) followed by no activity on SARS-CoV-2 and OC43 (Table 1). Cytotoxicity to Caco-2 showed a similar trend, CC_50_ being 88.7 μM and 61 μM after 24 and 72 h, respectively (Table S2). No relevant cytotoxicity was confirmed for compound **7** against Caco-2 cells at 24 h, while a slight increase was observed at 72 h (Table S2).

To assess the selectivity of the lead compounds against beta-coronaviruses, they were also tested for cytotoxicity and antiviral activity against an alternative, representative positive-sense single-stranded RNA virus, EVA-71.

Notably, none of the esters was able to reduce the cytopathogenicity induced by EV-A71 (Table S2), confirming the active selectivity against the betacoronaviruses tested in this study. 

### 3.1. Ester ***7*** Effect on Viral Yield

The antiviral activity of compound **7**, selected as lead, against the OC43 strain was confirmed in a virus yield reduction assay (YRA) against Vero-76, as reported in Figure 4B. The concentrations of 50, 20, 4 and 0.8 µM (not cytotoxic) were employed, and a significant dose-dependent reduction in the viral titer was observed. An interesting reduction in the OC43 titer (2 logs) was also detected at a low concentration of 4 µM (Figure 4B).

#### 3.1.1. Ester **7** Virucidal Activity Assay

To investigate the potential mechanisms of antiviral action, ester **7** was first subjected to a direct virus inactivation assay. The effect of **7** on hCoV-OC43 inactivation infectivity, before cell infection, was analyzed at 0 °C and 37 °C in an OC43 virucidal activity assay. The virus titers of samples treated with **7** did not significantly differ from those determined in untreated samples. Then, ester **7** did not exert its inhibitory effect by a direct inactivation of the hCoV-OC43 virion.

#### 3.1.2. Effect of Compound **7** on hCoV-OC43 Penetration into Pre-Treated Host Cells

To determine whether compound **7** was able to protect cells from hCoV-OC43 infection, a pre-treatment assay was then performed by incubating Vero-76 cell monolayers (2 h) with different concentrations of the conjugate (50, 20, 4, 0.8 μM). Dextran sulphate (DS), a broad-spectrum RNA/DNA-enveloped virus attachment inhibitor, was employed as a negative control. 

The unbound compounds were washed off, and the Vero-76 cells were then infected with OC43. The percentage of cell viability was determined after six days. 

**Figure 5:**
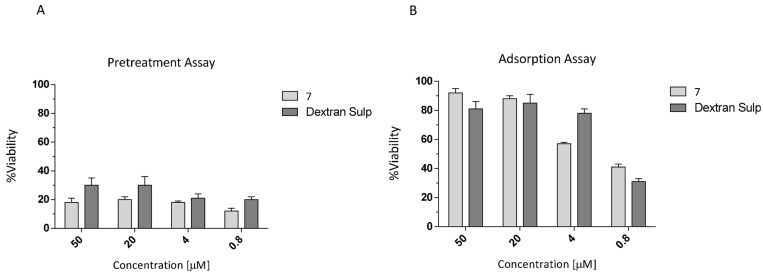
Cell Pretreatment Assay (**A**) and the dose-dependent inhibitory effect (**B**) of ester 7 on hCoV-OC43 adsorption. Data are expressed as means ± SD of at least three independent measurements.

The results reported in Figure 5A indicate that, under our experimental conditions, **7** failed to inhibit OC43 infection at the analyzed time point, as well as DS. These data show that pre-treatment with **7** does not protect monolayers from OC43 infection.

### 3.2. Adsorption Assay

We characterized the adsorption kinetic of hCoV-OC43 viral particles in the presence of ester **7**. We investigated whether **7** was able to interfere with the binding of the virus to target cells by incubating OC43 and cell monolayers at 4 °C in the presence of the compound. As reported in Figure 5B, **7** and Dextran sulphate (as positive control) were efficiently able to block OC43 viral binding under these conditions.

## 4. Discussion

Medicinal plants and their constituents are the basic source of many drugs and should be thoroughly studied to prove their safety and efficacy in human therapy, according to the World Health Organization (WHO) [37]. Flavonoids are phenolic compounds that are widespread in the plant world: more specifically, they are secondary metabolites produced by plants and are classified into different types, according to their chemical structure. 

Among the different flavonoids found in nature, the main subgroups are Anthoxanthins (flavanone and flavanol), flavans, flavanonols, flavanones, anthocyanidins, chalcones and isoflavonoids [10].

As these natural products have shown positive effects on human health, there has been an increasing effort to characterize their biological potential. In this context, we have carried out extensive research into flavonoids [36,37,38,39,40] and evaluated their efficacy as a complementary therapy to more specific antiviral compounds. More specifically, we have developed esters of cinnamic acids with quercetin to evaluate their cytotoxicity and potential antiviral activity in vitro.

Quercetin and cinnamic acids were evaluated alone and in parallel with their esters, against the coronavirus HCoV-229E, HCoV-OC43, and SARS-CoV-2. 

Recently, several articles have been published on quercetin and its ability to protect against coronaviruses [41]. 

Yue Zhu and co-authors have described the inhibitory effects of quercetin on the replication of HCoV-229E in Huh-7 cells with an estimated EC_50_ value of 4.88 μM [41] and on the Mpro activity of SARS-CoV-2 (IC_50_ = 6.79 μM).

Interestingly, quercetin and sinapic acid showed no anti-beta coronavirus activity in our assay. No inhibitory effect was observed when ferulic acid was evaluated in vitro, in a cell-based assay against HCoV-229E, HCoV-OC43 or SARS-CoV-2, and quercetin showed only weak activity against the alfa coronavirus 229E (EC_50_ = 51 μM).

Esters were evaluated for broad-spectrum anti-coronavirus activity, and **4**, **7** and **8** were found to be active against OC43, while **7** and **8** also showed activity against SARSCoV-2. None of them showed activity against the 229E strain or EVA71, employed as a representative positive RNA virus, suggesting a selective activity against beta coronaviruses.

Ester **7** showed an interesting antiviral activity (Table 1 and Figure 3), and it did not exert any toxic effects on various human cells, thus proving to be safe at the concentrations tested. Considering that it had the best safety profile, **7** was further investigated. When evaluated in a Yield reduction assay, it significantly reduced the viral titer in a dose-dependent manner (*p* < 0.05; *p* < 0.01).

Cells pre-treated with **7** showed no inhibition of OC43 replication, suggesting that the compound did not act directly on cell receptors; moreover, the treatment of virions did not determine the direct inactivation of the virion. Furthermore, we showed that **7** reduced the adsorption and penetration of OC43 into cell monolayers by the adsorption assay when compared to untreated samples. Data showed that it was able to block the attachment of OC43 to the host cell. We can hypothesize that 7 most likely prevents the viral envelope from binding to the cell membrane, thereby blocking subsequent stages of infection. 

The interpretation of these results must take into account the strengths and limitations of our study. The major strengths of our study include the characterization of new bioconjugates with broad beta coronavirus activity and the identification of ester **7**, which is endowed with anti-OC43 and SARS-CoV-2 activity.

However, compared to other studies, under our experimental conditions, quercetin did not show relevant antiviral activity in vitro, confirming its low or no activity in vivo and highlighting the need for further evidence on the biological properties of many products of natural origin, which are often controversially proposed as solutions for serious diseases. In the meantime, it is necessary to clarify the effective mechanism of antiviral action of these promising bioconjugates.

## 5. Conclusions

Historically, natural products, either in the form of pure compounds or as standardised plant extracts, have been an incentive in the creation of new drugs. Given the recent pandemic emergency, and the resurgence of the emergency of new and neglected pathogens, the results highlight the importance of natural products as promising resources as medication for humans in the pharmaceutical industry. In this study, we have analyzed the antiviral properties of esters of hydroxycinnamic acids with quercetin. The ester of synaptic acid 7 in particular turned out to be active against OC43 and SARS-CoV-2, showcasing an ability to induce a significant dose-dependent reduction in viral titre. 

Moreover, preliminary studies on the mode of action suggest a possible involvement of said esters in cellular viral penetration processes.

## Figures and Tables

**Table 1:** Cytotoxicity and antiviral activity of Quercetin and its esters with cinnamic acids against SARS-CoV-2, hCoV OC43, and hCoV-229E viruses in Normal Monkey kidney (Vero-76, supporting SARS-CoV-2 and OC4 3 replication) cells and Human Lung cancer (SK-MES-1, supporting 229E replication) cell line.

Compound	Structure	Vero-76	SARS-CoV-2	OC43	SK-MES-1	229E
		^a^ CC_50_	^c^ EC_50_		^b^ CC_50_	^d^ EC_50_
**1**	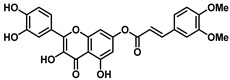	54.7	>54.7	>54.7	-	-
**2**	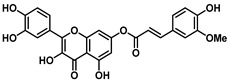	11.4	>11.4	>11.4	-	-
**3**	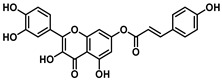	60	47.7 ± 0.32	>60	-	-
**4**	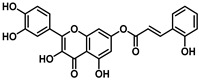	78	61	>78	-	-
**5**	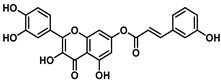	75	37	>75	-	-
**6**	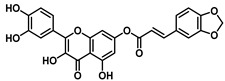	30	>30	2.4 ± 0.56	33	>33
**7**	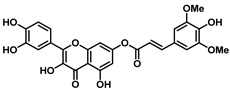	100	46.6	6.5 ± 0.7	>100	>100
**8**	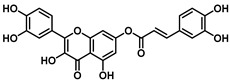	70	26	15	>100	>100
Quercetin	-	20	>20	>20	>100	51
Remdesivir	-	>100	1.6 ± 0.4	-	>100	5 ± 1
Hydroxychloroquine	-	60	-	1.9 ± 0.8	-	-

^a^ Compd concn (µM) required to reduce by 50% the viability of mock-infected Vero-76 cells (^a^ 120 h post-treatment), and SK-MES-1 (^b^ 48 h post-treatment) cells, as determined by the MTT method. ^c^ Compd concn (µM) required to reduce by 50% the plaque number of SARS-CoV-2 or required to achieve 50% protection of Vero-76 cells from OC43 (^d^), induced cytopathogenicity, as determined by the MTT method. ^d^ Compd concn (µM) required to achieve 50% protection of SK-MES-1 cells from 229E, induced cytopathogenicity, as determined by the MTT method.

## Data Availability

The data used to support the findings of this study are available from the corresponding author upon request.

## Supplementary Materials

The following supporting information can be downloaded at: https://www.mdpi.com/article/10.3390/v16050665/s1, Table S1: Cytotoxicity and antiviral activity of the cinnamic acids; Table S2: Cytotoxicity of Quercetin and ester **7** against human cell lines.

## References

[1] V’Kovski P., Kratzel A., Steiner S., Stalder H., Thiel V. (2021). Coronavirus biology and replication: Implications for SARS-CoV. Nat. Rev. Microbiol..

[2] https://www.who.int/publications/m/item/covid-19-epidemiological-update-16-february-2024.

[3] Baj J., Karakuła-Juchnowicz H., Teresiński G., Buszewicz G., Ciesielka M., Sitarz R., Forma A., Karakuła K., Flieger W., Portincasa P. (2020). COVID-19: Specific and Non-Specific Clinical Manifestations and Symptoms: The Current State of Knowledge. J. Clin. Med..

[4] Peng X.-L., Cheng J.-S., Gong H.-L., Yuan M.-D., Zhao X.-H., Li Z., Wei D.-X. (2021). Advances in the design and development of SARS-CoV-2 vaccines. Mil. Med. Res..

[5] Fuzo C.A., Martins R.B., Fraga-Silva T.F.C., Amstalden M.K., Canassa De Leo T., Souza J.P., Lima T.M., Faccioli L.H., Okamoto D.N., Juliano M.A. (2022). Celastrol: A lead compound that inhibits SARS-CoV-2 replication, the activity of viral and human cysteine proteases, and virus-induced IL-6 secretion. Drug Dev. Res..

[6] Elebeedy D., Elkhatib W.F., Kandeil A., Ghanem A., Kutkat O., Alnajjar R., Saleh M.A., El Maksoud A.I.A., Badawy I., Al-Karmalawy A.A. (2021). Anti-SARS-CoV-2 activities of tanshinone IIA, carnosic acid, rosmarinic acid, salvianolic acid, baicalein, and glycyrrhetinic acid between computational and in vitro insights. RSC Adv..

[7] Citarella A., Dimasi A., Moi D., Passarella D., Scala A., Piperno A., Micale N. (2023). Recent Advances in SARS-CoV-2 Main Protease Inhibitors: From Nirmatrelvir to Future Perspectives. Biomolecules.

[8] Citarella A., Moi D., Pedrini M., Pérez-Peña H., Pieraccini S., Dimasi A., Stagno C., Micale N., Schirmeister T., Sibille G. (2023). Synthesis of SARS-CoV-2 Mpro inhibitors bearing a cinnamic ester warhead with in vitro activity against human coronaviruses. Org. Biomol. Chem..

[9] Chaachouay N., Zidane L. (2024). Plant-Derived Natural Products: A Source for Drug Discovery and Development. Drugs Drug Candidates.

[10] Ullah A., Munir S., Badshah S.L., Khan N., Ghani L., Poulson B.G., Emwas A.-H., Jaremko M. (2020). Important Flavonoids and Their Role as a Therapeutic Agent. Molecules.

[11] Badshah S.L., Faisal S., Muhammad A., Poulson B.G., Emwas A.H., Jaremko M. (2021). Antiviral activities of flavonoids. Biomed. Pharmacother..

[12] Ahmad A., Kaleem M., Ahmed Z., Shafiq H. (2015). Therapeutic potential of flavonoids and their mechanism of action against microbial and viral infections—A review. Food Res. Int..

[13] Lalani S., Poh C.L. (2020). Flavonoids as Antiviral Agents for *Enterovirus A71* (*EV-A71*). Viruses.

[14] Adisakwattana S., Pongsuwan J., Wungcharoen C., Yibchok-Anun S. (2013). In Vitro Effects of Cinnamic Acid Derivatives on Protein Tyrosine Phosphatase 1B. J. Enzym. Inhib. Med. Chem..

[15] Lan J.S., Hou J.W., Liu Y., Ding Y., Zhang Y., Li L., Zhang T. (2017). Design, synthesis and evaluation of novel cinnamic acid derivatives bearing N-benzyl pyridinium moiety as multifunctional cholinesterase inhibitors for Alzheimer’s disease. J. Enzym. Inhib. Med. Chem..

[16] Theodosis-Nobelos P., Papagiouvannis G., Rekka E.A. (2023). Ferulic, Sinapic, 3,4-Dimethoxycinnamic Acid and Indomethacin Derivatives with Antioxidant, Anti-Inflammatory and Hypolipidemic Functionality. Antioxidants.

[17] Bian Y.-Y., Guo J., Majeed H., Zhu K.-X., Guo X.-N., Peng W., Zhou H.-M. (2015). Ferulic acid renders protection to HEK293 cells against oxidative damage and apoptosis induced by hydrogen peroxide. Vitr. Cell. Dev. Biol.-Anim..

[18] Lambruschini C., Demori I., El Rashed Z., Rovegno L., Canessa E., Cortese K., Grasselli E., Moni L. (2021). Synthesis, photoisomerization, antioxidant activity, and lipid Lowering effect of ferulic acid and feruloyl amides. Molecules.

[19] Montaser A., Huttunen J., Ibrahim S.A., Huttunen K.M. (2019). Astrocyte- targeted transporter-utilizing derivatives of ferulic acid can have multifunctional effects ameliorating inflammation and oxidative stress in the brain. Oxidative Med. Cell. Longev..

[20] Yin P., Zhang Z., Li J., Shi Y., Jin N., Zou W., Gao Q., Wang W., Liu F. (2019). Ferulic acid inhibits bovine endometrial epithelial cells against LPS-induced inflammation via suppressing NK-κB and MAPK pathway. Res. Vet. Sci..

[21] Ibitoye O.B., Ajiboye T.O. (2019). Ferulic acid potentiates the antibacterial activity of quinolone-based antibiotics against *Acinetobacter baumannii*. Microb. Pathog..

[22] Gao J., Yu H., Guo W., Kong Y., Gu L., Li Q., Yang S., Zhang Y., Wang Y. (2018). The anticancer effects of ferulic acid is associated with induction of cell cycle arrest and autophagy in cervical cancer cells. Cancer Cell Int..

[23] Antonopoulou I., Sapountzaki E., Rova U., Christakopoulos P. (2022). Ferulic acid from plant biomass: A phytochemical with promising antiviral properties. Front. Nutr..

[24] Liu S., Wei W., Li Y., Liu X., Cao X., Lei K., Zhou M. (2015). Design, synthesis, biological evaluation and molecular docking studies of phenylpropanoid derivatives as potent anti-hepatitis B virus agents. Eur. J. Med. Chem..

[25] Mao J.L., Wang L., Chen S.J., Yan B., Xun L.Y., Li R.C., Wang P.C., Zhao Q.T. (2023). Design, synthesis, antiviral activities of ferulic acid derivatives. Front. Pharmacol..

[26] Cui M.Y., Xiao M.W., Xu L.J., Chen Y., Liu A.L., Ye J., Hu A.X. (2020). Bioassay of ferulic acid derivatives as influenza neuraminidase inhibitors. Arch. Pharm..

[27] Zhou Z.Y., Xu J.Q., Zhao W.R., Chen X.L., Jin Y., Tang N., Tang J.Y. (2017). Ferulic acid relaxed rat aortic, small mesenteric and coronary arteries by blocking voltage- gated calcium channel and calcium desensitization via dephosphorylation of ERK1/2 and MYPT1. Eur. J. Pharmacol..

[28] Nićiforović N., Abramovič H. (2014). Sinapic Acid and Its Derivatives: Natural Sources and Bioactivity. Compr. Rev. Food Sci. Food Saf..

[29] Theodosis-Nobelos P., Kourti M., Tziona P., Kourounakis P.N., Rekka E.A. (2015). Esters of some non-steroidal anti-inflammatory drugs with cinnamyl alcohol are potent lipoxygenase inhibitors with enhanced anti-inflammatory activity. Bioorg. Med. Chem. Lett..

[30] Pavlíková N. (2023). Caffeic Acid and Diseases—Mechanisms of Action. Int. J. Mol. Sci..

[31] Chen Y., Li Z., Pan P., Lao Z., Xu J., Li Z., Zhan S., Liu X., Wu Y., Wang W. (2021). Cinnamic acid inhibits Zika virus by inhibiting RdRp activity. Antivir. Res..

[32] Amano R., Yamashita A., Kasai H., Hori T., Miyasato S., Saito S., Yokoe H., Takahashi K., Tanaka T., Otoguro T. (2017). Cinnamic acid derivatives inhibit hepatitis C virus replication via the induction of oxidative stress. Antivir. Res..

[33] Serreli G., Le Sayec M., Thou E., Lacour C., Diotallevi C., Dhunna M.A., Deiana M., Spencer J.P.E., Corana G. (2021). Ferulic Acid Derivatives and Avenanthramides Modulate Endothelial Function through Maintenance of Nitric Oxide Balance in HUVEC Cells. Nutrients.

[34] Sanna G., Farci P., Busonera B., Murgia G., La Colla P., Giliberti G. (2015). Antiviral properties from plants of the Mediterranean flora. Nat. Prod. Res..

[35] Ibba R., Carta A., Madeddu S., Caria P., Serreli G., Piras S., Sestito S., Loddo R., Sanna G. (2021). Inhibition of Enterovirus A71 by a Novel 2-Phenyl-Benzimidazole Derivative. Viruses.

[36] Zannella C., Giugliano R., Chianese A., Buonocore C., Vitale G.A., Sanna G., Sarno F., Manzin A., Nebbioso A., Termolino P. (2021). Antiviral Activity of Vitis vinifera Leaf Extract against SARS-CoV-2 and HSV-1. Viruses.

[37] https://www.who.int/news-room/feature-stories/detail/traditional-medicine-has-a-long-history-of-contributing-to-conventional-medicine-and-continues-to-hold-promise.

[38] DeToma A.S., Krishnamoorthy J., Nam Y., Lee H.J., Brender J.R., Kochi A., Lee D., Onnis V., Congiu C., Manfredini S. (2014). Synthetic Flavonoids, Aminoisoflavones: Interaction and Reactivity with Metal-Free and Metal-Associated Amyloid-β Species. Chem. Sci..

[39] Balboni G., Congiu C., Onnis V., Maresca A., Scozzafava A., Winum J.-Y., Maietti A., Supuran C.T. (2012). Flavones and structurally related 4-chromenones inhibit carbonic anhydrases by a different mechanism of action compared to coumarins. Bioorg. Med. Chem. Lett..

[40] Dinda B., Dinda M., Dinda S., Ghosh P.S., Das S.K. (2024). Anti-SARS-CoV-2, antioxidant and immunomodulatory potential of dietary flavonol quercetin: Focus on molecular targets and clinical efficacy. Eur. J. Med. Chem. Rep..

[41] Zhu Y., Scholle F., Kisthardt S.C., Xie D.Y. (2022). Flavonols and dihydroflavonols inhibit the main protease activity of SARS-CoV-2 and the replication of human coronavirus 229E. Virology.

