# Supplementary material for: Exploring the Antiviral Potential of Esters of Cinnamic Acids with Quercetin
Source: Viruses. 2024 Apr 24;16(5):665. doi: 10.3390/v16050665 (PMC11125646; doi:10.3390/v16050665)
Supplement: Supplementary file 1 [file viruses-16-00665-s001.zip › viruses-2958820-supplementary.pdf]

Exploring The Antiviral Potential of Esters of Cinnamic Acids with Quercetin

Valeria Manca[1#] Annalisa Chianese[2#], Vanessa Palmas[1], Federica Etzi[3], Carla Zannella[2], Davide Moi[6], Francesco Secci[4], Gabriele Serreli[5], Giorgia Sarais[6], Maria Vittoria Morone[2], Massimiliano Galdiero[2], Valentina Onnis[6], Aldo Manzin[1], and Giuseppina Sanna[1*]

[1]Dipartimento *di Scienze Biomediche, Microbiology and Virology Unit, Università degli Studi di Cagliari, Cittadella Universitaria, Monserrato, SP 8 Monserrato-Sestu Km 0,700, 09042, Monserrato, Italy;*
[2] *Department of Experimental Medicine, University of Study of Campania "Luigi Vanvitelli", Via Costantinopoli 16, 80138 Napoli, Italy;*
[3]Dipartimento *di Scienze Biomediche, Università degli Studi di Cagliari, Cittadella Universitaria, Monserrato, SP 8 Monserrato-Sestu Km 0,700, 09042, Monserrato, Italy;*
[4] *Dipartimento di Scienze Chimiche e Geologiche, Università degli Studi di Cagliari, Cittadella Universitaria, Monserrato, SP 8 Monserrato-Sestu Km 0,700, 09042, Monserrato, Italy;*
[5]Dipartimento *di Scienze Biomediche, Pathology Unit, Università degli Studi di Cagliari, Cittadella Universitaria, Monserrato, SP 8 Monserrato-Sestu Km 0,700, 09042, Monserrato, Italy;*
[6] *Dipartimento di Scienze della Vita e dell'Ambiente, Università degli Studi di Cagliari, Cittadella Universitaria, Monserrato, SP 8 Monserrato-Sestu Km 0,700, 09042, Monserrato, Italy;*

Supporting Information

1. Materials and methods

1.1 Chemistry

All commercially available solvents and reagents were used without further purification. ^{1}H NMR spectra were recorded using a Bruker Advance III HD 600 (Bruker, Bremen, Germany) at 600 MHz. The chemical shifts (δ) are reported in parts per million downfield from tetramethylsilane (TMS), which was used as an internal standard. The spectra were recorded in hexadeuteriodimethylsulphoxide (DMSO-d_6). Infrared spectra were recorded using a Nicolet iS10 spectrometer (Thermofisher Scientific, Whaltman, USA). The main bands are given in cm^{-1}. MS experiments were performed on an Agilent 6520 time-of-flight (TOF) MS LC/MS system (Agilent Technologies, USA). All compounds were analysed by ESI in negative mode. Mass hunter software was used for data acquisition and evaluation. Melting points (mp) were determined with a SMP1 Melting Point apparatus (Stuart Scientific, Stone, UK) and are uncorrected. All products reported showed ^{1}H NMR spectra in agreement with the assigned structures. The purity of the tested compounds was determined by combustion elemental analyses conducted by the Microanalytical Laboratory of the Chemistry Department of the University of Ferrara with an MT-5 CHN recorder elemental analyzer (Yanagimoto, Kyoto, Japan) and the values found were within 0.4% of theoretical values. The esters of hydroxycinnamic acid with quercetin were prepared under the following procedure. The hydroxycinnamic acid (1 mmol), 1-(3-dimethylaminopropyl)-3-ethylcarbodiimide hydrochloride (EDCI) (0.19 g, 1 mmol) and 1-hydrobenzotriazole (0.13 g, 1 mmol) were dissolved in anhydrous acetonitrile. The resulting mixture was stirred at r.t. for 30 minutes, then Quercetin (0.30 g, 1 mmol) was added. The mixture was stirred at r.t. for 72 hours. The solvent was removed under vacuum and the residue was washed sequentially with water (2 x 15 mL), saturated $NaHCO_3$ aqueous solution (2 x 15 mL), 10% citric acid (2 x 15 mL), and brine (2x10 mL). After drying the crude esters were recrystallized from EtOH to give the desired compounds.

Cell Culture

The Caco-2 cell line was obtained from ECACC (Salisbury, UK). Caco-2 cells are derived from human colorectal adenocarcinoma which, once reaching confluence, spontaneously differentiate into normal enterocytes. Dulbecco's modified Eagle's medium (DMEM) with low glucose and with L-Arginin, phosphate-buffered saline (PBS) without MgCl2 and CaCl2, fetal bovine serum (FBS) and penicillin/streptomycin 1X were obtained from Euroclone (Milano, Italy). Caco-2 cells were grown in T75 flasks until their confluence reached 80%, at 37 ◦C in a 5% CO2 humidified atmosphere in DMEM supplemented with 1% antibiotic/antimycotic solution (100 U/mL penicillin, 100 mg/mL streptomycin), and 10% FBS. At passage 21–40, cells were detached from flasks, collected, centrifuged and then seeded into 96-well plates at a concentration of 5 × 104 cells/mL for the MTT assay. Before the experiments, cells were kept in culture replacing the medium twice weekly.

MTT Assay

The MTT assay was performed as reported by Serreli et al., (2021) with slight modification. Briefly, cells were exposed to a range of concentrations of the compounds (0.8 – 200 μM, in serum free medium), or an equivalent volume of vehicle (MeOH) for the controls (0 μM), and incubated for 24 h and 72 h. After the treatments, the medium was replaced by 100 μL of MTT solution (0.5 mg/mL in supplemented growth media) and left for 4 h at 37 ◦C. The medium was removed, 100 μL of DMSO were added in each well and the absorbance was read at 570 nm by using a Multiskan Ex microplate reader (Thermo Fisher Scientific, Paisley, UK). Data were converted to % of cells viability as follows: % cell viability = Abs sample/Abs control × 100 and then expressed as CC_{50}.

2. Results

2.1 *Table S1*

Table S1. Cytotoxicity and antiviral activity of the cinnamic acids against SARS-CoV-2, hCoV OC43, and hCoV-229E viruses in Normal Monkey kidney (Vero-76) cells and Human Lung cancer (SK-MES-1) cell line.

Compound	R	Vero-76	SARS-CoV-2	OC43	SK-MES-1	229E
		$^{a}CC_{50}$ (μM)	$^{c}EC_{50}$ (μM)		$^{b}CC_{50}$ (μM)	$^{d}EC_{50}$ (μM)
**A1**	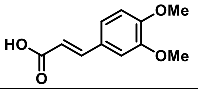	>100	>100	>100	-	-
**A2**	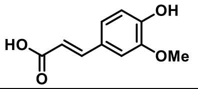	>100	>100	>100	-	-
**A3**	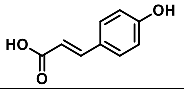	>100	>100	>100	-	-
**A4**	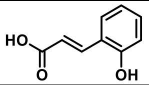	>100	>100	>100	-	-
**A5**	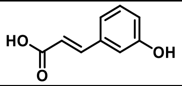	>100	>100	>100	-	-
**A6**	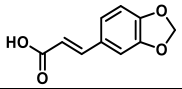					
**A7**	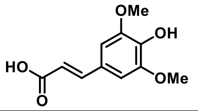	44±4	>44	>44	>100	>100
**A8**	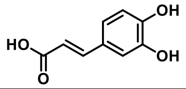	>100	>100	>100	>100	>100
**Remdesivir**	-	>100	1.6±0.4	-	>100	5±1
**Hydroxychloroquine**	-	60	-	1.9±0.8	-	-

Table S2. Cytotoxicity of Quercetin and ester 7 against human cell lines Caco-2, and Vero-76 at 24 and 72 hrs and antiviral activity against EV-A71.

**Compounds**	**^{a}Caco-2 (24h)/(72h)**	**^{b}Vero-76 (24h)/(72h)**	**EV-A71**
Quercetin	$^{c}CC_{50}$		$^{d}EC_{50}$
	88,7/61	>100/100	>100
7	>100/70	>100/>100	>100
Rupintrivir	-	>100	0.07

Data represent mean values ± SD for three independent determinations. For values where SD is not shown, variation among triplicate samples was less than 15%. [a]Human colorectal adenocarcinoma cells (as a model of the intestinal epithelial barrier); [b]Monkey kidney; [c]Compound concentration (μM) required to reduce Vero-76 cell proliferation by 50%, as determined by the MTT method; [d]Compound concentration (μM) required to achieve 50% protection from EVA71-induced cytopathogenicity, as determined by the MTT method.

2.2 Characterization data of final compounds 7

2-(3,4-dihydroxyphenyl)-3,5-dihydroxy-4-oxo-4*H*-chromen-7-yl(*E*)-3-(4-hydroxy-3,5-dimethoxyphenyl)acrylate **7**. Following the general procedure, the title compound was prepared starting from 3-methylphenylpiperazine. Yield 64% M.p. 203-205 °C. ^1H NMR (DMSO-d_6) δ 3.84 (s, 6H, OCH_3); 6.20 (m, 1H, Ar), 6.42 (m, 1H, Ar), 6.68 (s, 1H, Ar), 6.88 (s, 1H, Ar), 6.94 (d, J = 16.5 Hz, 1H, Ar); 7.15 (m, 1H, Ar); 7.45 (m, 1H, Ar); 7.76 (s, 1H, Ar), 7.78 (d, J = 16.22, 1H, Ar), 9.50 (bs, 1H, OH); 12.39 (bs, 1H, OH); 12.45 (s, 1H, OH); 12.48 (s, 1H, OH). ^{13}C NMR (DMSO-d_6) 175.1, 168.1, 159.7, 158.1, 156.2, 148.3, 147.9, 146.8, 145.6 (2C), 145.3, 138.9, 137.1, 127.8 (2C), 121.8, 121.6, 116.3 (2C), 115.9, 114.5, 110.7, 106.8, 103.8, 100.1, 56.2. IR (Nujol) 3298, 1651, 1596 cm^{-1}. ESI/MS (ESI^-) 507.1006.

References

1. Serreli G, Le Sayec M, Thou E, Lacour C, Diotallevi C, Dhunna MA, et al. Ferulic Acid Derivatives and Avenanthramides Modulate Endothelial Function through Maintenance of Nitric Oxide Balance in HUVEC Cells. Nutrients. 2021;13(6).
